# Compared with the intake of commercial vegetable juice, the intake of fresh fruit and komatsuna (*Brassica rapa* L. var. *perviridis*) juice mixture reduces serum cholesterol in middle-aged men: a randomized controlled pilot study

**DOI:** 10.1186/1476-511X-13-102

**Published:** 2014-06-24

**Authors:** Izumi Aiso, Hiroko Inoue, Yukiko Seiyama, Toshiko Kuwano

**Affiliations:** 1 Department of Food and Nutritional Sciences and Environmental Health Sciences, Graduate School of Integrated Pharmaceutical and Nutritional Sciences, University of Shizuoka, 52-1 Yada, Suruga-ku, 422–8526, Shizuoka, Japan; 2 School of Food and Nutritional Sciences, University of Shizuoka, Shizuoka, Japan

**Keywords:** Vegetable, Fruit, Serum cholesterol, Middle-aged men, Metabolic syndrome

## Abstract

**Background:**

Vegetables and fruits are rich in vitamins, minerals and, dietary fiber and contribute to the prevention and improvement of obesity and metabolic syndrome. However, inadequate intake of vegetable and fruit is a concern in Japan.

We therefore produced a juice mixture of fresh fruit and komatsuna (*Brassica rapa* L. var. *perviridis: B. rapa*) with the aim to investigate the effects of this juice mixture on anthropometric data, blood parameters, and dietary intake differences.

**Methods:**

This study was performed as a single blind and randomized controlled trial. Subjects were 16 men (mean age, 46.4 ± 7.1 years), and they were divided into two groups (control group and intervention group). The intervention group consumed the juice mixture of fresh fruit and *B. rapa*. The control group consumed commercial vegetable juice. Subjects consumed juice twice a day throughout the weekday, for 4 weeks. We prepared both juices with an equivalent energy balance.

**Results:**

Weight and body mass index (BMI) of the control group after 4 weeks were significantly increased compared with baseline values. Serum total cholesterol (T-Chol) and low-density lipoprotein cholesterol (LDL-Chol) of the intervention group after 4 weeks were significantly reduced compared with baseline values. Furthermore, intake of total vegetables and fruits were significantly increased compared with baseline values in both groups.

**Conclusions:**

Both vegetable juices contributed to improved intake of total vegetables and fruit. Compared with the intake of commercial vegetable juice, the intake of fresh fruit and *B. rapa* juice is highly effective in reducing serum cholesterol. Short-term intake of fresh fruit and *B. rapa* juice was shown to enhance cholesterol metabolism.

## Background

An increase in LDL-Chol concentration is a risk factor for arteriosclerosis. Arteriosclerosis is closely linked to increases in the incidence of coronary artery disease, stroke, and arteriosclerosis obliterans. A recent study indicates that a high LDL-Chol concentration increases the risk of arteriosclerosis, and a high T-Chol concentration increases the risk of coronary artery disease [1].

The consumption of vegetable and fruit improves lipid composition in blood. Guimares reported that intake of eggplant (*Solanum melongena*) infusion significantly reduced the blood concentrations of T-Chol, LDL-Chol, and apolipoprotein B in hypercholesterolemia subjects [2]. Futhermore, in a cohort study of 4466 subjects, Djoussé reported that consumption of fruits and vegetables is inversely related to LDL-Chol [3].

Moreover, previous studies show an association between fruit and vegetable consumption and coronary artery disease, ischemic heart disease, and cerebrovascular disease. Increasing the consumption of fruit and vegetables reduces the risk of coronary artery disease, stroke, and hypertension [4]. Compared with people who consume vegetables and fruits once or less a day, those who consume vegetables and fruits thrice or more a day have lower stroke incidence, stroke mortality, ischemic heart disease mortality, and cardiovascular disease mortality [5].

High consumption of vegetables and fruits may have effects on blood lipid metabolism and reduce the risk of diseases stemming from arteriosclerosis.

In Japan, “Healthy Japan 21” was developed as a prophylaxis of life-style related diseases by Health, Labour and the Welfare Ministry in 2000 [6]. Healthy Japan 21 promotes a vegetable intake of more than 350 g/day, green and yellow vegetable intake of more than 120 g/day, and fruit intake of 200 g/day. However, these goals may still be unattainable in all life stages [6].

To improve the current situation, we need to take some drastic measures. With such an aim, we produced fresh of fruit and *B. rapa* juice mixture as a means of providing easy intake of vegetable and fruit.

There are reports that daily intake of commercial vegetable juice increases dietary vegetable intake [7,8]. However, reports of intervention studies using fresh fruit and vegetable juice are few [9-11].

We conducted an intervention study to investigate whether daily intake of a juice mixture from fresh fruit and *B. rapa* would affect anthropometric data, blood parameters, and dietary intake.

## Results

### Characteristics of subjects

Characteristics of the study subjects are shown in Table 1. The mean anthropometric data of the study subjects did not exceed the reference value for metabolic syndrome. However, BMI and waist circumference tended to be moderately high.

**Table 1:** Characteristics of subjects

	**Mean ± S.D.**
Age (years)	46.6 ± 7.1
Height (cm)	172.3 ± 5.7
Weight (kg)	73.3 ± 16.8
Body fat percentage (%)	23.2 ± 8.1
Body mass index (kg/m2)	24.6 ± 5.2
Waist Circumference (cm)	87.4 ± 13.6
Systolic blood pressure (mmHg)	126 ± 16
Diastolic blood pressure (mmHg)	79 ± 12

Data shows mean ± S.D.

### **Nutrient components of commercial vegetable juice and fresh fruit and *B. rapa *** **juice mixture**

Nutrient components of both juices are shown in Table 2. Nutrition components of commercial vegetable juice (100 g) were as follows: energy, 48 kcal; potassium, 173 mg; and β-carotene, equivalents 2628 μg. In contrast, nutrition components of fresh fruit and *B. rapa* juice mixture (100 g) were as follows: energy, 44 kcal; potassium, 180 mg; and β-carotene equivalents, 360 μg. Volume of protein, fat, carbohydrate, sodium, iron, folate, vitamin C, and dietary fiber are shown in Table 2.

**Table 2:** **Nutrient components of commercial vegetable juice and fresh fruit and *B. rapa *** **juice mixture**

	**Commercial vegetable juice**^**※1)**^	**Fresh fruit and *B. rapa *** **juice mixture**^**※2)**^
Energy (kcal/100 g)	48	44
Protein (g/100 g)	0.4	0.7
Fat (g/100 g)	0	0
Carbohydrate (g/100 g)	12.3	10.3
Sodium (mg/100 g)	43	12
Potassium (mg/100 g)	173	180
Iron (mg/100 g)	0.2	0.2
β-carotene equivalent (μg/100 g)	2628	360
Folate (μg/100 g)	9	8
Vitamin C (mg/100 g)	9	35
Dietary fiber (g/100 g)	1.0	0.5

※1) Nutritional labeling of commercial vegetable juice.

※2) Analysis values by Japan Inspection Association of Food and Food Industry Environment.

*Abbreviation*: *B. rapa* Brassica rapa L. var. perviridis.

### Anthropometric data

Anthropometric data of both groups are shown in Table 3. Weight and BMI after 4 weeks in the control group were significantly increased compared with baseline values (weight: 70.7 ± 14.1 vs 71.9 ± 13.8 kg, *p* = 0.017; BMI: 23.8 ± 4.9 vs 24.2 ± 4.8 kg/m^2^, *p* = 0.042). In contrast, there were no significant differences in the intervention group.

**Table 3:** Change of anthropometric data

	**Control group (n = 8)**			**Intervention group (n = 8)**		
	**Baseline**	**After 4 weeks**		**Baseline**	**After 4 weeks**	
	**Mean ± S.D.**	**Mean ± S.D.**	**p**	**Mean ± S.D.**	**Mean ± S.D.**	**p**
Height (cm)	172.3 ± 6.7	172.3 ± 6.7	0.735	172.4 ± 4.9	172.4 ± 4.7	0.611
Weight (kg)	70.7 ± 14.1	71.9 ± 13.8	0.017*	76.0 ± 19.7	76.3 ± 19.4	0.362
BFP (%)	24.3 ± 9.5	24.9 ± 9.6	0.484	22.1 ± 7.0	23.1 ± 7.5	0.069
BMI (kg/m2)	23.8 ± 4.9	24.2 ± 4.8	0.042*	25.4 ± 5.6	25.5 ± 5.6	0.259
Waist circumference	86.6 ± 12.9	86.5 ± 12.8	0.932	88.3 ± 15.2	88.2 ± 15.2	0.326
Systolic blood pressure (mmHg)	125 ± 13	128 ± 15	0.327	126 ± 19	133 ± 17	0.050
Diastolic blood pressure (mmHg)	79 ± 12	80 ± 10	0.147	79 ± 14	82 ± 12	0.237

Data shows mean ± S.D.

*Abbreviations*: *BFP* Body fat percentage, *BMI* Body mass index.

Baseline vs After 4 weeks, Wilcoxon signed rank test, *p < 0.05.

### Blood parameters

Blood parameters of both groups are shown in Table 4. T-Chol and LDL-Chol after 4 weeks in the intervention group were significantly decreased compared with baseline values (T-Chol: 220 ± 28 vs 211 ± 27 mg/dL, *p* = 0.017; LDL-Chol: 143 ± 27 vs 134 ± 23 mg/dL, *p* = 0.017).

**Table 4:** Change of blood parameter

	**Control group (n = 8)**			**Intervention group (n = 8)**		
	**Baseline**	**After 4 weeks**		**Baseline**	**After 4 weeks**	
	**Mean ± S.D.**	**Mean ± S.D.**	**p**	**Mean ± S.D.**	**Mean ± S.D.**	**p**
Glucose (mg/dL)	95 ± 6	96 ± 6	0.359	100 ± 7	96 ± 10	0.106
Hemoglobin A1c (%)	5.3 ± 0.3	5.3 ± 0.3	0.589	5.7 ± 0.8	5.6 ± 0.4	0.526
Triacylglycerol (mg/dL)	121 ± 82	160 ± 118	0.080	122 ± 47	121 ± 49	1.000
Free fatty acid (μEq/L)	586 ± 526	451 ± 132	0.779	497 ± 184	379 ± 54	0.069
T-Chol (mg/dL)	210 ± 38	210 ± 33	0.833	220 ± 28	211 ± 27	0.017*
HDL-Chol (mg/dL)	50 ± 12	51 ± 15	0.570	53 ± 15	53 ± 13	0.799
LDL-Chol (mg/dL)	136 ± 36	127 ± 23	0.262	143 ± 27	134 ± 23	0.017*
Insulin (μIU/mL)	8.72 ± 11.40	10.80 ± 14.77	0.161	7.39 ± 8.42	9.42 ± 13.03	0.327
Vitamin C (μg/mL)	2.38 ± 1.14	1.82 ± 0.83	0.441	1.76 ± 0.49	1.80 ± 0.44	0.726
Leptin (pg/mL)	236 ± 301	214 ± 255	0.401	129 ± 99	161 ± 185	1.000
Adiponectin (μg/mL)	13.2 ± 13.8	13.3 ± 13.9	1.000	8.2 ± 5.9	8.3 ± 5.7	0.866
HOMA-IR	2.10 ± 2.83	2.69 ± 3.87	0.655	1.92 ± 2.38	2.44 ± 3.71	0.144
Arteriosclerotic Index	3.49 ± 1.58	3.55 ± 1.87	0.779	3.48 ± 1.59	3.16 ± 1.05	0.327

Data shows mean ± S.D.

*Abbreviation*: *T-Chol* Total cholesterol, *HDL-Chol* High-density lipoprotein cholesterol, *LDL-Chol* Low-density lipoprotein cholesterol, *HOMA-IR* Homeostasis model of assessment of insulin resistance.

Baseline vs After 4 weeks, Wilcoxon signed rank test, *p < 0.05.

### Energy and nutrition intake

Energy and nutrition intake of both groups are shown in Table 5. Potassium intake after 4 weeks in both groups were significantly increased compared with baseline values (control group: 2,550 ± 1,105 vs 3,049 ± 1,068 mg, *p* = 0.012; intervention group: 2,815 ± 978 vs 3,486 ± 766 mg, *p* = 0.017). β-carotene equivalent intake after 4 weeks in the intervention group was significantly increased compared with the baseline value (3,815 ± 2,357 vs 4,795 ± 1,881 μg, *p* = 0.036). Magnesium intake after 4 weeks in the intervention group tended to increase compared with the baseline value (286 ± 69 vs 312 ± 64 mg, *p* = 0.069).

**Table 5:** Change of energy and nutrition intakes

	**Control group (n = 8)**			**Intervention group (n = 8)**		
	**Baseline**	**After 4 weeks**		**Baseline**	**After 4 weeks**	
	**Mean ± S.D.**	**Mean ± S.D.**	**p**	**Mean ± S.D.**	**Mean ± S.D.**	**p**
Energy (kcal)	1,829 ± 401	1,955 ± 460	0.327	2,190 ± 293	2,247 ± 364	0.401
Protein (g)	73.1 ± 24.8	74.9 ± 26.7	0.484	77.3 ± 17.0	76.7 ± 16.7	0.889
Fat (g)	52.3 ± 15.0	50.1 ± 16.1	0.484	69.8 ± 14.7	61.0 ± 15.9	0.069
Carbohydrate (g)	248.0 ± 52.5	291.9 ± 77.0	0.327	288.0 ± 26.3	325.7 ± 54.0	0.025*
Sodium (mg)	4,251 ± 1,233	4,576 ± 1,519	0.123	4,339 ± 918	4,564 ± 868	0.401
Potassium (mg)	2,550 ± 1,105	3,049 ± 1,068	0.012*	2,815 ± 978	3,486 ± 766	0.017*
Calcium (mg)	611 ± 249	572 ± 283	0.401	610 ± 190	608 ± 159	0.674
Magnesium (mg)	266 ± 99	278 ± 101	0.263	286 ± 69	312 ± 64	0.069
Phosphorus (mg)	1,105 ± 392	1,126 ± 426	0.575	1,172 ± 282	1,173 ± 258	0.889
Iron (mg)	8.4 ± 3.5	8.9 ± 3.4	0.123	9.0 ± 2.6	9.8 ± 2.5	0.093
Zinc (mg)	8.7 ± 2.8	8.9 ± 2.9	0.484	9.1 ± 1.8	8.9 ± 1.9	0.833
Manganese (mg)	3.5 ± 1.1	3.5 ± 1.1	0.401	3.6 ± 0.9	3.4 ± 1.0	0.208
β-carotene equivalent (μg)	3,926 ± 2,674	3,945 ± 1,639	0.674	3,815 ± 2,357	4,795 ± 1,881	0.036*
Retinol equivalent (μg)	753 ± 555	808 ± 471	0.779	929 ± 459	855 ± 370	0.674
α-tocopherol (mg)	7.0 ± 2.5	7.6 ± 2.4	0.161	8.9 ± 2.9	9.7 ± 2.4	0.161
Vitamin C (mg)	101.6 ± 50.9	188.0 ± 67.0	0.012*	121.2 ± 67.3	223.9 ± 46.9	0.012*
Saturated fat (g)	13.6 ± 3.9	13.2 ± 5.0	0.674	18.7 ± 3.5	16.4 ± 3.9	0.093
Monounsaturated fat (g)	18.4 ± 5.4	17.7 ± 5.4	0.575	25.0 ± 5.3	21.5 ± 6.1	0.093
Polyunsaturated fat (g)	13.5 ± 4.0	12.4 ± 4.0	0.263	17.2 ± 4.1	14.9 ± 3.8	0.069
Cholesterol (mg)	417 ± 205	424 ± 193	0.889	434 ± 127	441 ± 153	0.779
Total dietary fiber (g)	12.2 ± 5.2	11.6 ± 4.0	0.484	13.6 ± 3.8	14.1 ± 3.9	0.484
Soluble dietary fiber (g)	3.1 ± 1.5	3.2 ± 1.3	0.673	3.5 ± 1.1	3.9 ± 1.1	0.093
Insoluble dietary fiber (g)	8.7 ± 3.5	8.1 ± 2.6	0.263	9.6 ± 2.5	9.6 ± 2.6	1.000
Sodium chloride equivalent (g)	10.8 ± 3.1	11.6 ± 3.8	0.123	10.9 ± 2.3	11.5 ± 2.2	0.401

Data shows mean ± S.D.

Baseline vs After 4 weeks, Wilcoxon signed rank test, *p < 0.05.

Vitamin C intake after 4 weeks in both groups were increased compared with baseline values (control group: 101.6 ± 50.9 vs 188.0 ± 67.0 mg, *p* = 0.012; intervention group: 121.2 ± 67.3 vs 223.9 ± 46.9 mg, *p* = 0.012).

### Food intake

Food intakes of both groups are shown in Table 6. Total vegetable and fruit intake after 4 weeks in both groups were increased compared with baseline values (total vegetable value of control group: 246.0 ± 149.0 vs 324.6 ± 121.3 g, *p* = 0.012; total vegetable value of intervention group: 280.7 ± 168.3 vs 396.9 ± 121.5 g, *p* = 0.012; fruits value of control group: 70.1 ± 63.0 vs 438.3 ± 181.3 g, *p* = 0.017; fruits value of intervention group: 106.2 ± 91.8 vs 524.0 ± 44.2 g, *p* = 0.012). Furthermore, green and yellow vegetables intake after 4 weeks in both groups were increased compared with baseline values (control group: 105.5 ± 71.8 vs 177.8 ± 60.4 g, *p* = 0.012; intervention group: 108.1 ± 78.5 vs 225.2 ± 54.6 g, *p* = 0.012).

**Table 6:** Change of food intake

	**Control group (n = 8)**			**Intervention group (n = 8)**		
	**Baseline**	**After 4 weeks**		**Baseline**	**After 4 weeks**	
	**Mean ± S.D.**	**Mean ± S.D.**	**p**	**Mean ± S.D.**	**Mean ± S.D**	**p**
Cereals (g)	459.1 ± 125.5	490.8 ± 190.9	0.735	456.9 ± 102.6	428.8 ± 129.7	0.310
Potatoes (g)	53.2 ± 38.6	47.0 ± 23.2	0.600	52.3 ± 46.0	48.4 ± 16.3	0.600
Nuts and pulses (g)	87.0 ± 57.8	72.1 ± 57.3	0.237	79.3 ± 34.8	73.4 ± 37.9	0.600
Total vegetables (g)	246.0 ± 149.0	324.6 ± 121.3	0.012*	280.7 ± 168.3	396.9 ± 121.5	0.012*
Green and yellow vegetables (g)	105.5 ± 71.8	177.8 ± 60.4	0.012*	108.1 ± 78.5	225.2 ± 54.6	0.012*
Other vegetables (g)	140.6 ± 78.9	146.8 ± 74.8	0.484	172.6 ± 95.1	171.7 ± 68.9	0.674
Fruits (g)	70.1 ± 63.0	438.3 ± 181.3	0.017*	106.2 ± 91.8	524.0 ± 44.2	0.012*
Fish and shellfish (g)	75.4 ± 56.9	74.9 ± 53.5	0.866	71.1 ± 31.5	75.3 ± 31.5	0.674
Meat (g)	65.6 ± 30.3	82.6 ± 44.5	0.263	77.8 ± 29.1	72.5 ± 27.1	0.398
Eggs (g)	51.5 ± 32.0	53.2 ± 27.5	0.798	41.7 ± 24.7	48.6 ± 26.7	0.310
Dairy products (g)	191.8 ± 103.1	156.4 ± 129.0	0.176	174.7 ± 82.8	150.4 ± 55.0	0.176
Fat and oil (g)	10.4 ± 6.5	10.3 ± 6.0	0.735	14.6 ± 5.3	12.9 ± 4.3	0.575
Confectionery (g)	28.8 ± 23.0	20.1 ± 17.7	0.263	73.6 ± 42.1	64.7 ± 34.0	0.463
Beverage preference (g)	621.4 ± 325.3	508.8 ± 337.3	0.069	850.0 ± 406.0	790.7 ± 281.9	0.401

Data shows mean ± S.D.

Baseline vs After 4 weeks, Wilcoxon signed rank test, *p < 0.05.

## Discussion

We performed an intervention pilot study on the effects of short-term intake of either a juice mixture of fresh fruit and *B. rapa* or commercial vegetable juice in middle-aged men. The aim of the present study was to compare the effects of a juice mixture of fresh fruit and *B. rapa* with those of commercial vegetable juice on anthropometric data, blood parameters, and dietary intake.

T-Chol and LDL-Chol after 4 weeks in the intervention group were significantly decreased compared with baseline values. However, T-Chol and LDL-Chol after 4 weeks in the control group were unchanged compared with baseline values. Short-term intake of juice mixture of fresh fruit and *B. rapa* is thought to enhance cholesterol metabolism. Subjects with hypercholesterolemia who were given two cans of a beverage of mixed green vegetable and fruit beverage (containing broccoli and cabbage for 12 weeks) had reduced LDL-Chol after 3 weeks compared with baseline values [12]. Our results showed a similar result, which infers that reductions in LDL-Chol may involve increases in fecal bile acid by S-methylcysteine sulfoxide contained in *Brassica* vegetables [12].

Potassium and Vitamin C intake after 4 weeks in both groups were increased compared with baseline values. β-carotene equivalent intake after 4 weeks in the intervention group was increased compared with baseline values. These findings are related to reports on mineral and lipids metabolism from previous studies. Potassium increases activity of LPL through high insulin concentrations [8,13,14]. Magnesium is the essential mineral for the enzyme that is involved in lipid metabolism, capable of decreasing T-Chol concentrations [15,16]. Magnesium intake after 4 weeks in the intervention group tended to increase compared with the baseline value in the present study. Furthermore, β-carotene reduces the activity of 3-hydroxy-3-methylglutaryl coenzyme A reductase and inhibits the synthesis of cholesterol [17,18].

Antioxidant vitamins are related to lipid metabolism. LDL-Chol binds to the LDL receptor and is translocated into the cell. However, when LDL-Chol is oxidized, receptor binding affinity is decreased. Antioxidant vitamins prevent oxidation of LDL-Chol and supports lipid metabolism [11]. Vitamin C intake after 4 weeks in both groups were significantly increased compared with baseline values in the present study. Furthermore, β-carotene equivalent intake after 4 weeks in the intervention group was increased compared with the baseline value. These results show that intake of fresh fruit and *B. rapa* juice mixture increased antioxidant vitamins.

Weight and BMI after 4 weeks in the control group were significantly increased compared with baseline values in the present study. However, there was no difference in dietary energy after 4 weeks compared with the baseline value. We used brief and self-administered diet history questionnaires (BDHQ) as a dietary survey method in our study. BDHQ has been used and validated in many studies [19-21]. However, BDHQ is not as accurate as weighed dietary records. Hence, it was not clear whether dietary changes in the present study caused the increases in weight and BMI in the control group.

Commercial juice contains 7 times more carotene than the juice mixture comprising fresh fruit and *B. rapa*. However, β-carotene intake in the control group did not change from baseline. BDHQ does not enable the identification of the types of vegetables included in the vegetable juice. Therefore, the fresh fruit and *B. rapa* juice mixture and commercial juice were calculated as vegetable juice. For these reasons, we were not able to confirm an effect of the β-carotene equivalent of the commercial juice using the dietary survey.

The limitation of the present study is the short-term intervention. We also did not have a control group that did not drink juice. In future, we will perform a long-term intervention of fresh fruit and *B. rapa* juice mixture, and also examine the effects on lipid metabolism.

## Conclusions

We performed an intervention pilot study to compare the effects of a short-term intake of fresh fruit and *B. rapa* juice mixture with those of commercial vegetable juice in middle-aged men.

Regular consumption of vegetable juices contributed to increased intake of total vegetables and fruits. Interestingly, compared with the intake of commercial vegetable juice, the intake of fresh fruit and *B. rapa* juice mixture is highly effective in reducing serum cholesterol.

Short-term intake of fresh fruit and *B. rapa* juice mixture most likely enhances cholesterol metabolism.

## Methods

### Preparation of fresh fruit and *B. rapa* juice mixture and commercial juice

**Figure 1:**
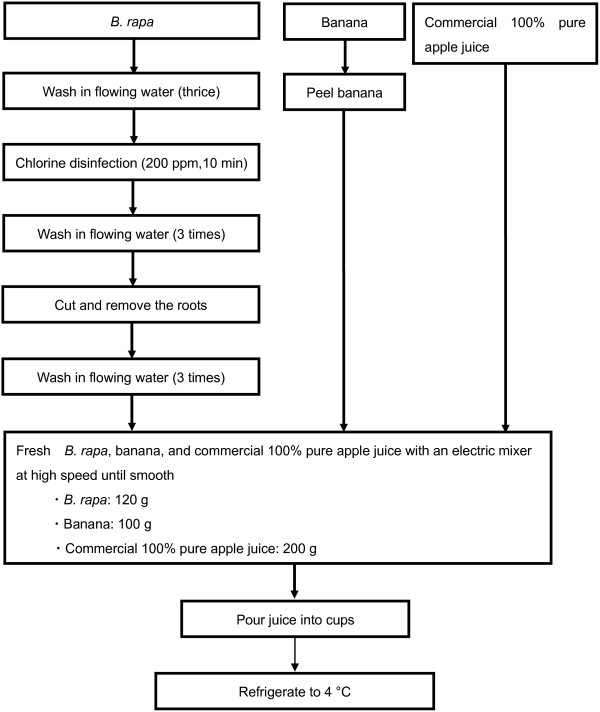
**Preparation of fresh fruit and *B. rapa *juice mixture.** The ingredients of the juice include *B. rapa* (120 g), banana (100 g), and 100% pure, commercial apple juice (200 g). The ingredients were blended on high speed with an electric mixer (Vitamix Model No. VM0111, VITA-MIX CORP., USA) until smooth (1 min, 37,000 rpm). Preparation of the juice mixture followed the guidelines from the “sanitary management of large scale cooking facilities manual [22].

Figure 1 shows the protocol adopted for the preparation of fresh fruit and *B. rapa* juice mixture. The ingredients of the juice are *B. rapa* (120 g), banana (100 g), and commercial 100% pure apple juice (200 g). We beat *B. rapa*, banana and commercial 100% pure apple juice with an electric mixer (Vitamix Model No. VM0111, VITA-MIX CORP., USA) at high speed until smooth (1 min., 37,000 rpm). Preparation of this juice mixture was on the basis of the “sanitary management of large scale cooking facilities manual [22]”. Vegetable juice of the control group was a commercialized product. We prepared fresh fruit and *B. rapa* juice mixture every day. The commercial juice is a blend of several fruits (grape, banana, apple, lemon, and acerola) and various vegetables (carrots, sweet potatoes, lettuce, red pepper, kidney beans, kale, green pepper, Chinese cabbage, broccoli, celery, asparagus, pumpkin, komatsuna, *Angelica keiskei*, parsley, watercress, cabbage, radish, spinach, and Japanese honewort). It was produced by ITO EN, Ltd (trade name: ITO EN Jujitsu Yasai Banana Mix, Ito En, Ltd, Tokyo, Japan).

Nutrient components of the fresh fruit and *B. rapa* juice mixture were analyzed by Japan Inspection Association of Food and Food Industry Environment. Nutrient components of the commercial vegetable juice were noted as per the nutritional labeling on the juice package.

### Subjects

Subjects were 16 worker men with a mean age of 46.4 ± 7.1 years. The subjects volunteered after we described the study in great detail. The predominant work of the subjects was desk work. The physical activity level of the subjects were described as low. They were divided into two groups (control and intervention groups). One person had Grave’s disease in the study, who was taking medication. However, no effect on the blood marker was found, regardless of his exclusion. Other than this subject, no other subjects took medication. This study was conducted after receiving approval from the University of Shizuoka ethics committee (No. 24–13). In this study written informed consent was obtained from all the subjects.

### Study design

This study was performed for 4 weeks as a single blind, randomized controlled trial from October 30th to November 27th, 2012.

Both groups consumed juice twice a day (at 10:00 a.m. and 3:00 p.m.) every weekday. The intervention group consumed freshly prepared fruit and *B. rapa* juice mixture (210 g). The control group consumed a commercial vegetable juice (ITOEN JUJITSU YASAI BANANA MIX, ITO EN, LTD, Tokyo, Japan) (200 g) at the same time. The fruit and *B. rapa* juice mixture of the intervention group and the commercial vegetable juice of the control group were of equivalent energy content. The subjects were unaware of which juice they consumed.

### Anthropometric measurements and blood sampling

Subjects fasted from 20:00 of the night before body measurements and blood collection. Height, weight, body fat percentage (BFP), waist circumference, and blood pressure were measured the following morning between 08:00 and 10:00. Body weight and BFP were measured with a body fat analyzer (TBF-215; Tanita, Tokyo, Japan). Waist circumference was measured with a tape measure at the level of the navel. Blood pressure was measured with a digital automatic sphygmomanometer (HEM-5001; OMRON, Kyoto, Japan). Waist circumference and blood pressure were measured twice each, and the mean was calculated. Blood was collected between 07:00 and 10:00.

Blood for plasma vitamin C analysis was collected in vacuum blood collection tubes containing sodium fluoride and dipotassium EDTA, and blood for other tests was collected in vacuum blood collection tubes containing heparin. After collection, blood collection tubes were immediately inverted and mixed, and those for other tests were left at room temperature for 30 min after collection. Collection tubes were then centrifuged for 15 min at 4°C and 3000 rpm. The plasma portion for vitamin C analysis and the serum portion for other tests were then added to microtubes and stored at -80°C until analysis. Analyses of total protein, high-density lipoprotein cholesterol (HDL-Chol), LDL-Chol, triacylglycerol, free fatty acids, and hemoglobin A_1c_ were outsourced to SRL (Tokyo, Japan). Serum leptin was analyzed with a Human Leptin (Highly Sensitive) Assay Kit (Immuno-Biological Laboratories, Gunma, Japan). Serum adiponectin was analyzed with an Adiponectin Assay kit (Otsuka Pharmaceutical Co., Ltd, Japan). Plasma vitamin C was analyzed by the α,α’-Dipyridyl method [23,24].

### Food and nutrition survey

The food and nutrition survey used was the BDHQ. The BDHQ is a 4-page fixed-portion questionnaire that asks about the consumption frequency of selected foods, but not about portion size, to estimate the dietary intake of 58 food and beverage items during the preceding month [19-21].

### Statistical analysis

A normality test (Using Shapiro–Wilk test) was performed before conducting comparisons on measures before and after the intervention. When normal distribution was found, we used a paired t-test for comparisons before and after the intervention. Furthermore, a Wilcoxon signed-rank test was performed when normal distribution was not found. Data were analyzed using SPSS for Windows version 15.0 J computer software (SPSS Japan Inc, Tokyo, Japan). Values of *p* < 0.05 were accepted as significant. All values in the text and tables are represented as the mean ± standard deviation.

## Abbreviations

*B. rapa*: *Brassica rapa* L. var. *perviridis*; T-Chol: Total cholesterol; LDL-Chol: Low-density lipoprotein cholesterol; BMI: Body mass index; BDHQ: Brief-type self-administered diet history questionnaire; BFP: Body fat percentage; EDTA: Ethylenediaminetetraacetate; HDL-Chol: High-density lipoprotein cholesterol; HOMA-IR: Homeostasis model assessment insulin resistance.

## Competing interests

The authors declare that they have no competing interest.

## Authors’ contributions

All authors managed the study. IA, HI and YS conducted blood analysis and dietary assessment. IA, HI, YS and TK undertook statistical analysis. IA, HI and TK drafted the manuscript. All authors read and approved the final manuscript.

## Authors’ information

Laboratory of Nutrition Education, Department of Food and Nutritional Sciences and Environmental Health Sciences, Graduate School of Integrated Pharmaceutical and Nutritional Sciences, University of Shizuoka, Shizuoka 422–8526, Japan.
